# Postoperative Pulmonary Complications and Perioperative Strategies: A Systematic Review

**DOI:** 10.7759/cureus.38786

**Published:** 2023-05-09

**Authors:** João Lusquinhos, Mafalda Tavares, Fernando Abelha

**Affiliations:** 1 Anesthesiology, Centro Hospitalar Universitário de São João, Porto, PRT; 2 Occupational Health, Centro Hospitalar Universitário de São João, Porto, PRT; 3 Surgery, Faculdade de Medicina da Universidade do Porto, Porto, PRT

**Keywords:** hospital length of stay, re-intubation, postoperative mechanical ventilation, postoperative noninvasive ventilation, postoperative pulmonary complications

## Abstract

The occurrence of postoperative pulmonary complications (PPCs) is frequently observed and has been linked to elevated levels of morbidity and mortality, which have adverse effects on both clinical and financial outcomes in healthcare settings. This systematic review aims to present the evidence that supports our comprehension of PPCs and emphasize the circumstances that necessitate the use of postoperative noninvasive ventilation (PNIV) or re-intubation with postoperative mechanical ventilation (POMV). A search was conducted on the National Library of Medicine's Pubmed database and Cochrane Library until November 29, 2020, to find published reports of randomized control trials (RCTs) that assessed postoperative pulmonary complications. Data related to the prevalence of PPCs and the use of PNIV, POMV, and length of hospital stay were extracted from all the studies. For the analysis, a total of 13 studies involving 6,609 patients were included, and out of these, four RCTs reported statistically significant results. The use of protective lung ventilation (PLV) with low tidal volume and positive end-expiratory pressure (PEEP) during intraoperative ventilation, along with pressure-controlled (PCV) ventilation, as well as the postoperative ventilation strategy of continuous positive airway pressure (CPAP) combined with standard oxygen therapy were the only techniques that demonstrated a clear reduction in the incidence of PPCs.

Furthermore, the use of PLV with low tidal volume and PEEP and intraoperative mechanical ventilation with a vital capacity maneuver followed by 10 cm H2O of PEEP were found to decrease the requirement for postoperative noninvasive ventilation. CPAP with standard oxygen therapy was the only intervention that reduced the need for reintubation. Various ventilation strategies are available for both intraoperative and postoperative periods with the goal of decreasing the need for postoperative noninvasive ventilation (PNIV) or re-intubation with postoperative mechanical ventilation (POMV).

## Introduction and background

The occurrence of postoperative pulmonary complications (PPCs) is frequently observed and has been linked to elevated levels of morbidity and mortality, which have adverse effects on both clinical and financial outcomes in healthcare settings [1,2]. The incidence of PPC varies depending on the clinical treatment setting, the type of surgery, and the definition of PPC used, ranging from 1% to 23% [3,4]. Several studies have shown pulmonary complications to be more common than cardiac complications and more frequent following abdominal and thoracic surgery [5,6]. Significant morbidity associated with PPC results from a higher length of hospital stay due to the increased necessity for admission to intensive care units, with a possible need for re-intubation and ventilatory support [4]. Therefore, the cost burden associated with PPCs is substantial to the point where preventing these pulmonary complications will be of enormous interest to the healthcare system [1,4].

PPC is defined as any pulmonary abnormality occurring in the postoperative period that produces an identifiable disease or dysfunction that is clinically significant and adversely affects the clinical course after surgery [7,8]. This leads to a wide variety of disorders ranging from self-limited alterations, such as mild degrees of atelectasis or bronchospasm, to more severe conditions with substantial morbidity and mortality, such as pneumonia or respiratory failure. The conventional definition of PPC includes conditions such as atelectasis, bronchospasm, pneumonia, and exacerbation of chronic lung disease [9]. However, the list can be extended to include acute upper airway obstruction, complications from obstructive sleep apnea, pleural effusions, chemical pneumonitis, pulmonary edema, hypoxemia, and tracheal laceration or rupture [9]. 

Apart from PPC multifactorial etiology, they are also associated with countless preoperative, intraoperative, and postoperative risk factors. Miskovic and Lumb categorized these into patient-related, procedure-related, and laboratory testing that could be further detailed into non-modifiable and modifiable risk factors, such as age, smoking, comorbidities, chronic lung disease, and type of surgery. Consistent etiologic factors include decreased functional residual capacity (FRC) and total lung capacity (TLC) that result in ventilation-perfusion mismatch and hypoxemia [4,5].

Whenever postoperative respiratory dysfunction occurs, such as hypoxemia induced by atelectasis, the first choice method for ventilatory support to prevent re-intubation is noninvasive ventilation (NIV) [2,10]. NIV is expected to increase gas lung volume, improve gas exchange and reduce atelectasis. Still, if respiratory failure persists and acute lung injury is shown, invasive mechanical ventilation (MV) may be required. Reduction of postoperative complications requires preoperative identification of at-risk patients and subsequent modification of these risk factors. Thereby, given the financial impact, the prevention of PPC is of extreme importance for the healthcare system. Yet, it is imperative to understand risk factors, improve intraoperative management and identify interventions that may impact PPC [5,6].

This systematic review aims to present the evidence that supports our comprehension of PPCs and emphasize the circumstances that necessitate the use of postoperative noninvasive ventilation (PNIV) or re-intubation with postoperative mechanical ventilation (POMV).

## Review

Methods

We performed a systematic qualitative review following the guidelines of the Preferred Reporting Items for Systematic Reviews and Meta-Analyses statement (PRISMA).

Systematic Research

A search was conducted on the National Library of Medicine's Pubmed database and Cochrane Library until November 29, 2020, to find published reports of randomized control trials (RCTs) that assessed postoperative pulmonary complications. Keywords and MeSH terms were used individually and in various combinations, creating the final query found in the appendices (appendix A). No attempt to identify additional studies not found by the primary search methods was made by reviewing the reference lists from identified studies. No search was performed for unpublished studies. This initial search yielded 1.090 randomized controlled trials.

Query used in our research: (((( "Anesthesia, General" [Mesh] OR anesthesia[tw] OR "general anesthesia" [tw] OR "general anesthesia" [tw]) AND ("Postoperative Complications" [Mesh] OR postoperative[tw] OR "postoperative complication*" [tw] OR postop[tw]) AND ("Lung" [Mesh] OR "Lung Diseases" [Mesh] OR "Pulmonary Heart Disease" [Mesh] OR "Pulmonary Disease, Chronic Obstructive" [Mesh] OR "Respiratory System" [Mesh] OR "Pulmonary Atelectasis" [Mesh] OR "Respiration, Artificial" [Mesh] OR ventilation[tw] OR "mechanical ventilation" [tw] OR "pulmonary" [tw] OR "respiratory system" [tw] OR "lung*" [tw] OR "lung disease*" [tw] OR "pulmonary disease*" [tw] OR "pulmonary atelectasis" [tw] OR atelectasis[tw]))) AND (randomizedcontrolledtrial[Filter])).

Inclusion and Exclusion Criteria

The study's inclusion and exclusion criteria were determined before the systematic search. We included all RCTs published until November 29, 2020. No language restriction was used. Studies regarding postoperative complications that require PNIV or re-intubation with POMV were also included.

Selection of Included Studies

**Figure 1:**
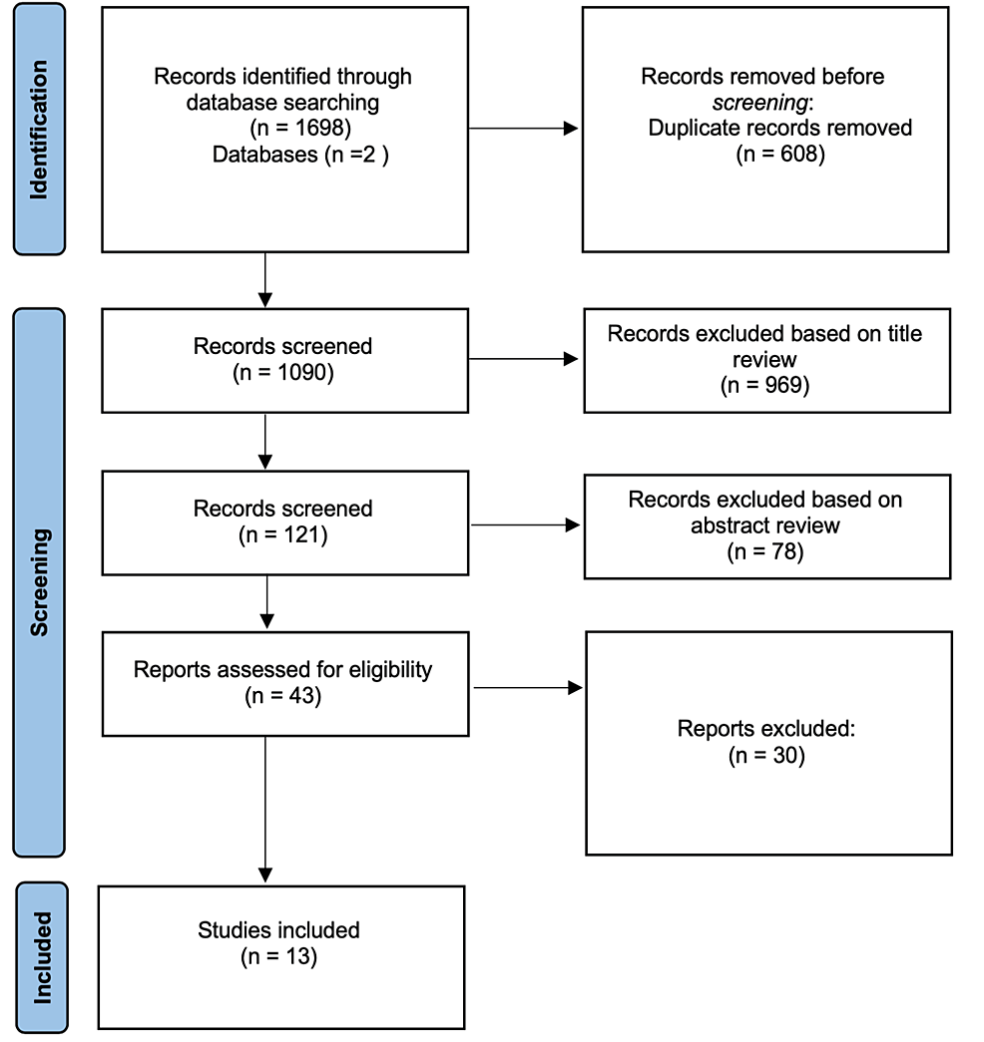
Flowchart of the literature review process.

Two authors independently evaluated the titles of the 1698 articles obtained by the initial search, and 608 were removed before screening because they were duplicates. At the first step of the screening, 969 articles (evaluated by the same two authors) were excluded at this phase based on the title review. Disagreements on including the articles were resolved by discussion among the evaluators. If an agreement could not be reached, the dispute was resolved with the help of a third investigator. The third investigator was blinded regarding the evaluation of the first two authors. Then, we conducted a second screening by evaluating the abstracts of the remaining articles, and of those, 78 were excluded for not meeting the inclusion criteria. Afterward, we excluded 30 articles based on full-text review plus 4 articles we could not reach full-text access, the remaining 13 articles used in this systematic review. Figure 1 illustrates the process of screening and selection of the information.

Quality Assessment

**Figure 2:**
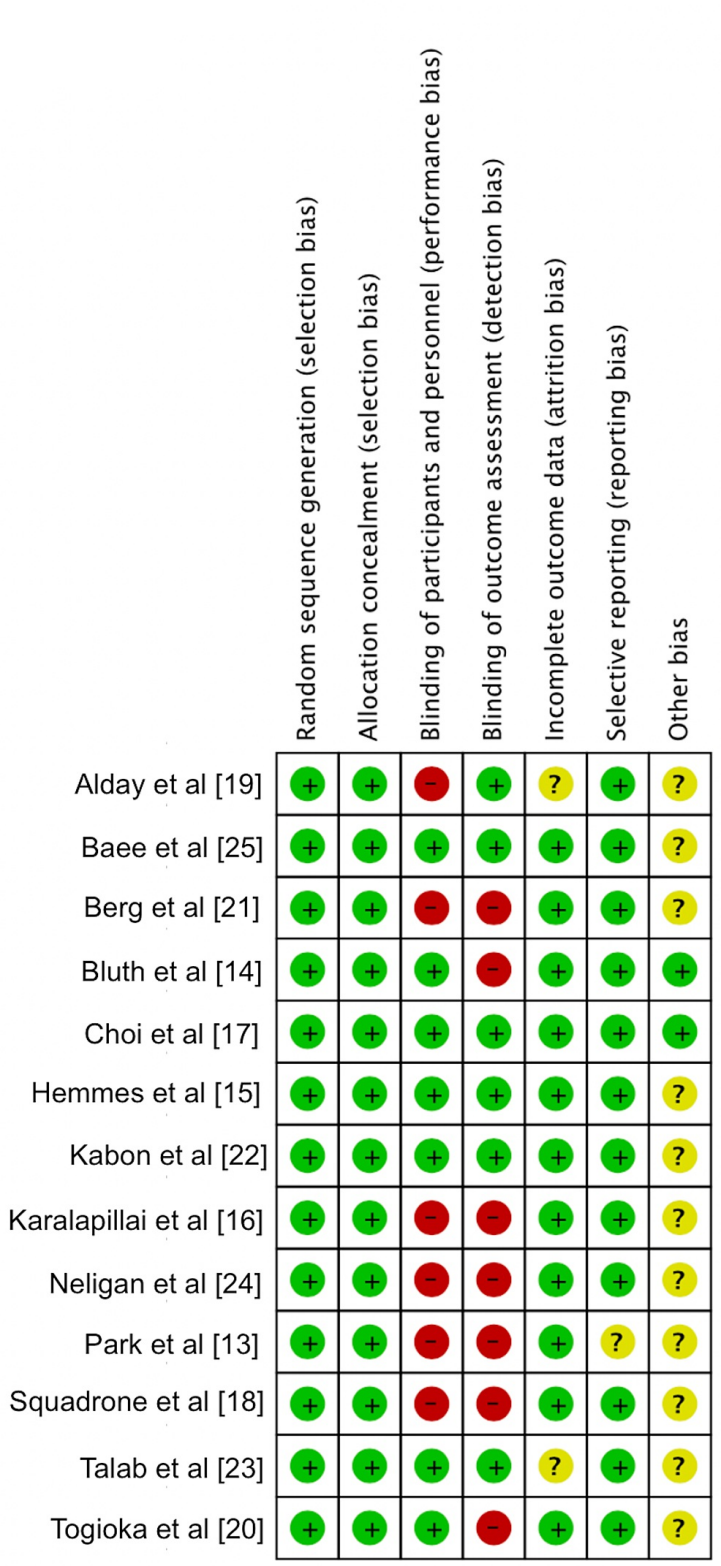
Risk of bias

The risk of bias was also independently assessed by the two reviewers, and data was collected using the Cochrane Collaboration tool for assessing the risk of bias, which covers random sequence generation, allocation concealment, blinding of participants and personnel, blinding of outcome assessment, incomplete outcome data, selective outcome reporting and other bias [11]. The six items on the list can be rated as "high risk", "low risk," or "unclear risk". We computed graphic representations of potential bias within studies (Figure 2).

Data Extraction

Two authors independently evaluated the full manuscripts of all included RCTs and performed data extraction. Discrepancies were resolved by discussion between the two investigators. If an agreement could not be reached between the two investigators, the decision was made by a third investigator. Data extracted from trials included the number of patients, age of the patients, country, study period, type of anesthesia, type of surgery, intervention group, and results, such as incidence of PPC, the incidence of PNIV, the incidence of POMV, and length of hospital stay.

Definition of Relevant Outcome Data

The primary outcome was the incidence of PPC. They were defined using the European Perioperative Clinical Outcomes (EPCO) definitions and other clinical conditions not included in EPCO guidelines [12]. These include respiratory infection, respiratory failure, pleural effusion, atelectasis, pneumothorax, bronchospasm, aspiration pneumonitis, pneumonia, acute respiratory distress syndrome (ARDS), tracheobronchitis, pulmonary edema, exacerbation of pre-existing lung disease and pulmonary embolism. We also recorded secondary outcomes of interest: the incidence of PNIV, the incidence of POMV, and the length of hospital stay (LOS).

Results

Study Characteristics

In total, thirteen studies that involved 6609 patients were included in our systematic review. The basic characteristics are presented in Table 1. Six included studies were related to intraoperative ventilation strategies, two were related to postoperative ventilation strategies, four were related to neuromuscular blockade, and one evaluated crystalloid versus colloid administration. Among these thirteen studies, ten were used on our primary outcome - incidence of PPC, and twelve studies were used on secondary outcomes - incidence of PNIV, the incidence of POMV, and length of hospital stay. Moreover, six studies were conducted in Europe, four in the United States of America (USA), two in Asia, and one in Australia. Adjustment for potential confounders varied across studies, and most risk appraisals were adjusted for age, gender, sex, body mass index (BMI), baseline oxygen saturation, and ARISCAT scale for postoperative risk of pulmonary complications.

**Table 1:** Characteristics of the studies included in the systematic review No., number; NS, non-specified; RCT, randomized control trial.

Study	No. Of patients	Country	Study Period	Design
Park et al. [13].	39	USA	2012-2014	RCT
Bluth et al. [14].	1976	USA	2014-2018	RCT
Hemmes et al. [15].	894	Netherlands	2014	RCT
Karalapillai et al. [16].	1206	Australia	2015-2019	RCT
Choi et al. [17].	46	Korea	2014-2018	RCT
Squadrone et al. [18].	209	Italy	2002-2003	RCT
Alday et al. [19].	126	Spain	2015-2016	RCT
Togioka et al. [20].	200	USA	2017-2018	RCT
Berg et al. [21].	691	Denmark	NS	RCT
Kabon et al. [22].	1056	Austria	2006-2016	RCT
Talab et al. [23].	66	Saudi Arabia	NS	RCT
Neligan et al. [24].	40	USA	NS	RCT

Postoperative Pulmonary Complications

The correlations between each study and the incidence of PPC are summarized in Table 2. Regarding our enrollment criteria, 10 studies were included. We divided the studies into four subgroups based on the main intervention: intraoperative ventilation strategies, postoperative ventilation strategies, neuromuscular blockage, and crystalloid vs. colloid administration.

**Table 2:** Secondary Outcomes CPAP, Continuous Positive Airway Pressure; FiO2, Fraction of inspired oxygen; LHS, Length of hospital stay; NA, non applicable; NMB, Neuromuscular block; n.s., non significant; PEEP, Positive end expiratory pressure; PNIV, Postoperative noninvasive ventilation; POMV, Postoperative mechanical ventilation; VCM, Vital Capacity Maneuver; ZEEP, Zero end-expiratory pressure.

Study	PNIV	POMV	LOS
INTRAOPERATIVE VENTILATION STRATEGIES			
Park et al. [13].	42% vs. 13% (p=0.049) (Group R vs. Group P, respectively).	0% on both groups. (NA) (Group R vs. Group P, respectively).	10 days on both groups. (p=0.499) (Group R vs. Group P, respectively).
Karalapillai et al. [16].	2.5% vs. 2.4% (p=0.92) (Low tidal volume group vs. Conventional tidal volume group, respectively).	2.5% vs. 2.4% (p=0.92) (Low tidal volume group vs. Conventional tidal volume group, respectively).	8.3 days vs. 7.9 days (p=0.40) (Low tidal volume group vs. Conventional tidal volume group, respectively).
Hemmes et al.[15].	NA	4% vs. 5% (p=0.74) (Higher PEEP group vs. Lower PEEP group, respectively).	10 days on both groups. (p=0.24) (Higher PEEP group vs. Lower PEEP group, respectively).
Talab et al. [23].	4.5%* vs. 16.7% vs. 26.3%(*p<0.05) (PEEP10 group vs. PEEP5 group vs. ZEEP group, respectively).	NA	NA
Choi et al. [17].	NA	10% vs. 1% (p=0.005) (Control group vs. CPAP group, respectively).	17 days vs. 15 days (p=0.10) (Control group vs. CPAP group, respectively).
POSTOPERATIVE VENTILATION STRATEGIES			
Squadrone et al. [18].	NA	10% vs. 1% (p=0.005) (Control group vs. CPAP group, respectively).	17 days vs. 15 days (p=0.10) (Control group vs. CPAP group, respectively).
NEUROMUSCULAR BLOCKADE			
Alday et al [19].	0% vs. 3% (NA) (Neostigmine group. vs. Sugammadex group, respectively)	3% vs. 3% (NA) (Neostigmine group. vs. Sugammadex group, respectively)	11.4 days vs. 12.9 days (NA) (Neostigmine group. vs. Sugammadex group, respectively)
Togioka et al [20].	NA	NA	4.5 days vs. 4.0 days (p=0.42) (Neostigmine group. vs. Sugammadex group, respectively)
Berg et al. [21].	10% vs. 10.9% (NA) (Pancuronium group vs. Atracurium or Vecuronium group, respectively)	NA	NA
Baete et al. [25].			NA
CRYSTALLOID VS. COLLOID			
Kabon et al. [22].	NA	NA	7 days for both groups (p=0.45)

Intraoperative Ventilation Strategies

Park et al. study compared the effect of intraoperative conventional ventilation combined with alveolar recruitment maneuvers (Group R) with protective lung ventilation strategy (Group P) on postoperative pulmonary complications [13]. PPC occurred less frequently in group P than in group R (14% vs. 47%, respectively; p=0.023) and postoperative atelectasis (14% vs. 42%, respectively; p=0.023). Pneumonia only occurred in Group R (5%; p=0.023). In a study by Bluth et al. [14], the effectiveness of an intraoperative mechanical ventilation strategy with a higher level of positive end-expiratory pressure (PEEP) and alveolar recruitment maneuvers were compared to a lower level of PEEP without alveolar recruitment maneuvers in reducing the incidence of postoperative pulmonary complications (PPCs). This study found that within the first five days following surgery, the occurrence of PPCs was more frequent in the low-level PEEP group than in the high-level PEEP group, although the difference was not statistically significant (23.6% vs. 21.3%, respectively; p=0.23). The most common pulmonary complication observed was mild respiratory failure, which was also more prevalent in the low-level PEEP group (15.6% vs. 13.7%, respectively; p=0.22). Additionally, atelectasis was more frequently observed in the low-level PEEP group compared to the high-level PEEP group (5.6% vs. 4.4%, respectively; p=0.25). Hemmes et al. [15] designed a study to test the hypothesis that a ventilation strategy with a high positive end-expiratory pressure plus recruitment maneuvers protects against postoperative pulmonary complications. PPC occurred in 40% of the patients in the higher PEEP group and 39% in the lower PEEP group (p=0.84). Atelectasis did not differ between groups (12% vs. 12%, respectively; p=0.90). A study conducted by Karalapillai et al. [16] aimed to investigate whether using a low-tidal volume ventilation strategy would reduce the incidence of postoperative pulmonary complications (PPCs) in adult patients undergoing major surgery, compared to conventional-tidal volume ventilation while receiving the same level of positive end-expiratory pressure (PEEP). The study found that the incidence of PPCs within the first seven days post-surgery was not significantly different between the low-tidal-volume group (38%) and the conventional-tidal-volume group (39%) (p=0.64). The most common PPC observed in both groups was atelectasis, which occurred in 24.7% and 24.9% of patients in the low-tidal-volume and conventional-tidal-volume groups, respectively (p=0.93). In a study conducted by Choi et al. [17], the researchers investigated the differences in airway mechanics and postoperative pulmonary complications (PPCs) based on the mode of mechanical ventilation used. The study found that the incidence of PPCs was three times higher in the volume-controlled ventilation group (39.1%) compared to the pressure-controlled ventilation group (13.0%) (p=0.044).

Postoperative Ventilation Strategies

Squadrone et al. [18] aimed to evaluate the effectiveness of continuous positive airway pressure (CPAP) compared to standard oxygen therapy in preventing the need for intubation and mechanical ventilation in patients who developed acute hypoxemia after undergoing elective major abdominal surgery. They verified that all the cases of pneumonia occurred within the first week after surgery, affecting 2% of the patients treated with oxygen plus CPAP versus 10% of the patients treated with standard oxygen therapy (p=0.02).

Neuromuscular Blockade

Alday et al. [19] study tested the hypothesis that reversal neuromuscular block with sugammadex would result in less postoperative pulmonary dysfunction than reversal with neostigmine. They also evaluated the incidence of postoperative atelectasis and pneumonia as secondary outcomes and verified that twenty-four hours after surgery, 74% of the patients in the neostigmine group had detectible atelectasis versus 66% in the sugammadex group (p>0.25), and that incidence of postoperative pneumonia did not differ between groups (1% vs. 1%, respectively; p-value not applicable).

Togioka et al. hypothesized that sugammadex would reduce the incidence of postoperative pulmonary complications compared to neostigmine [20]. They observed that the incidence of PPC was lower in the sugammadex group than in the neostigmine group (33% vs. 40%, respectively; p=0.30). Furthermore, atelectasis occurred less frequently in the sugammadex group compared with the neostigmine group (19% vs. 25%, respectively; p=0.31); pneumonia incidence as well as respiratory complications incidence were similar in both groups (3% vs. 2%, respectively; p=1.0 / 4% vs. 2%, respectively; p=0.68).

Berg et al. [21] undertook a study to compare the incidence of postoperative pulmonary complications following the use of neuromuscular blocking drugs, namely long-acting pancuronium and the intermediate-acting drugs atracurium and vecuronium; the authors also determined whether residual neuromuscular blockade was a risk factor for the development of PPC. The overall incidence of PPCs was 6.7%. The long-acting group had a greater incidence of PPC than the intermediate-acting group (8.3% vs. 11.7%, respectively; n.s.). In addition, the incidence of postoperative neuromuscular block, defined as a train-of-four (TOF) ratio1 < 0.7, wasn't statistically different between groups (26% vs. 11%, respectively, n.s.); however, the long-acting group had a greater incidence of PPC and neuromuscular residual block when compared with the intermediate-acting group (16.9%* vs. 4.2%, respectively; *p<0.02).

Crystalloid vs. Colloid Administration

Kabon et al. [23] tested the hypothesis of a 30-day composite of postoperative complications and compared the administration of colloids versus crystalloids as intraoperative fluids. They observed that the overall incidence of PPCs was similar in both groups (4.49% vs. 3.25%, respectively; n.s.).

Secondary Outcomes: Postoperative Noninvasive Ventilation, Postoperative Mechanical Ventilation and Length of Hospital Stay

The correlations between each study and the incidence of PNIV and/or POMV and LOS are summarized in Table 3. Regarding our enrollment criteria, twelve studies were included. We divided the studies into four subgroups based on the main intervention: intraoperative ventilation strategies, postoperative ventilation strategies, neuromuscular blockade, and crystalloid vs. colloid administration.

**Table 3:** Primary outcomes CPAP, Continuous Positive Airway Pressure; FiO2, fraction of inspired oxygen; n.s., non significant; PPC, postoperative pulmonary complications; PEEP, positive end expiratory pressure.

Study	Interventions	Endpoints	Incidences
INTRAOPERATIVE VENTILATION STRATEGIES			
Park et al. [13].	Group R - conventional ventilation with alveolar recruitment maneuver. VS. Group P - protective lung ventilation (low tidal volume with PEEP 5 cm H_2_O).	Incidence of postoperative pulmonary complications. Incidence of postoperative desaturation.	Overall postoperative pulmonary complications: 47% vs. 14%. (p=0.023) Atelectasis: 42% vs. 14%. (p=0.023) Pneumonia: 5% vs. 0%. (p=0.023) (Group R vs. Group P, respectively).
Bluth et al. [14].	High level of PEEP group - PEEP level of 12 cm H_2_O with alveolar recruitment maneuvers. VS. Low level of PEEP group - PEEP level of 4 cm H_2_O.	Incidence of postoperative pulmonary complications.	Overall postoperative pulmonary complications: 21.3% vs. 23.6%. (p=0.23) Mild respiratory failure: 13.7% vs. 15.6%. (p=0.22) Atelectasis: 4.4% vs. 5.6%. (p=0.25) Pneumonia: 1% vs. 1%. (p>0.99) (High level of PEEP group vs. Low level of PEEP group, respectively).
Hemmes et al. [15].	Higher PEEP group - high level of PEEP (12 cm H_2_O) with recruitment manoeuvres. VS. Lower PEEP group - a low level of pressure (≤ 2 cm H_2_O) without recruitment manoeuvres.	Incidence of postoperative pulmonary complications.	Postoperative pulmonary complications: 40% vs. 39%. (p=0.84) Atelectasis: 12% vs. 12%. (p=0.90) Pneumonia: 16% vs. 17%. (p=0.58) (Higher PEEP group vs. Lower PEEP group, respectively).
Karalapillai et al. [16].	Low tidal volume group – tidal volume of 6mL/kg predicted body weight. VS. Conventional tidal volume group - tidal volume of 10 mL/kg predicted body weight . All patients PEEP at 5 cm H_2_O.	Incidence of postoperative pulmonary complications.	Postoperative pulmonary complications: 38% vs. 39%. (p=0.64) Atelectasis: 24.7% vs. 24.9%. (p=0.93) (Low tidal volume group vs. Conventional tidal volume group, respectively).
Choi et al. [17].	Volume controlled ventilation group. VS. Pressure controlled ventilation group.	Incidence of postoperative respiratory complications.	Postoperative respiratory complications: 39.1% vs. 13.0%. (p=0.044) (Volume controlled group vs. Pressure controlled group, respectively).
POSTOPERATIVE VENTILATION STRATEGIES			
Squadrone et al. [18].	Control group - treated for 6 hours with oxygen through a Venturi mask at an FiO_2_ of 0.5. VS. CPAP group - treated with oxygen at an FiO_2_ of 0.5 plus a CPAP of 7.5 cm H_2_O.	Incidence of pneumonia.	Pneumonia: 10% vs. 2%. (p=0.02) (Control group vs. CPAP group, respectively).
NEUROMUSCULAR BLOCKADE			
Alday et al. [19].	Neostigmine group. VS. Sugammadex group.	Incidence of atelectasis. Incidence of pneumonia.	Atelectasis 1h after surgery: 39% vs. 30%. (p > 0.25) Atelectasis 24h after surgery: 74% vs. 66%. (p > 0.25) Pneumonia: 1% vs. 1%. (NA) (Neostigmine group. vs. Sugammadex group, respectively)
Togioka et al. [20].	Pancuronium group VS. Atracurium or Vecuronium group.	Incidence of postoperative pulmonary complications.	Postoperative pulmonary complications: 40% vs. 33%. (p=0.30) Pneumonia: 2% vs. 3%. (p=1.0) Atelectasis: 25% vs. 19%. (p=0.31) Respiratory complication: 2% vs. 4%. (p=0.68) (Neostigmine group. vs. Sugammadex group, respectively)
Berg et al. [21].	Pancuronium group VS. Atracurium or Vecuronium group.	Incidence of postoperative pulmonary complications.	Overall postoperative pulmonary complications: 6.7%. (n.s.) Postoperative pulmonary complications: 8.3% vs. 5.6%. (n.s.) Postoperative pulmonary complications with residual block (TOF ratio 3 < 0.7): 16.9%* vs. 4.2%. (*p< 0.02) (Pancuronium group. vs. Atracurium or Vecuronium group, respectively)
CRYSTALLOID VS. COLLOID			
Kabon et al. [22].	Crystalloid group - goal- directed crystalloid administration (lactated Ringer’s solution). VS. Colloid group - goal- directed colloid administration (hydroxyethyl starch 6%).	Incidence of postoperative morbidity.	Overall pulmonary complications (overall - 30 day): 4.49% vs. 3.25%. (n.s.) Pulmonary complications before discharge: 3.18% vs. 3.05%. (n.s.) (Crystalloid group vs. Colloid group, respectively)

Park et al. [13] study evaluated the length of hospital stay and recorded the need for postoperative noninvasive ventilation or mechanical ventilation. They verified that LOS did not differ between groups (10 days in both groups; p=0.499), but the incidence of PNIV was higher in the conventional ventilation with alveolar recruitment maneuver group when compared with the protective lung ventilation group (42% vs. 13%, respectively; p=0.049). None of the patients needed POMV (0% in both groups; NA).

Karalapillai et al. [16] likewise evaluated unplanned requirements for postoperative mechanical ventilation, continuous positive airway pressure, or noninvasive ventilation and patients' LOS. They found that 2.5% of the patients in the low tidal volume group required unplanned noninvasive or invasive ventilation compared with 2.4% of the patients in the conventional tidal volume group (p=0.92). LOS was 8.3 days in the low tidal volume group versus 7.9 days in the conventional tidal volume group (p=0.40).

Hemmes et al. [15] also analyzed the need for new or continued postoperative mechanical ventilation and patients' LOS. Nor POMV or LOS differed between groups. The incidence of POMV in the higher PEEP group was 4% compared with 5% in the lower PEEP group (p=0.74), and LOS was 10 days in both groups (p=0.24).

Talab et al. [23] study evaluated the safety and efficacy of vital capacity maneuvers followed by different levels of PEEP used to prevent postoperative lung atelectasis and also recorded the need for PNIV. Postoperatively, only 4.5%* of the patients in the PEEP10 group needed oxygen from a nonrebreathing O_2_ mask (FiO_2_ 100%) versus 16.7% in the PEEP5 group and 26.3% in the zero-positive end-expiratory pressure (ZEEP) group (*p<0.05). Choi et al. [17] also concluded that patients' LOS did not differ between groups (10 days in the volume-controlled group vs. 9 days in the pressure-controlled group; p=0.293).

As previously mentioned, Squadrone et al. [18] study evaluated the effectiveness of CPAP compared with standard oxygen therapy treatment in preventing the need for intubation, mechanical ventilation, and patients' LOS. They observed that the intubation rate in patients treated with oxygen and CPAP was lower than in those treated with oxygen alone (1% vs. 10%, respectively; p=0.005). The mean LOS was similar between groups (15 days in patients treated with CPAP versus 17 days in patients treated with oxygen; p=0.10).

Neligan et al. [24] aimed to determine whether immediate post-extubation CPAP, administered using the Boussignac system, improves lung function compared with CPAP started in the recovery room and evaluated reintubation incidence as a secondary outcome. They reported that none of the patients enrolled in the study were reintubated at any time.

Alday et al. [19] also documented hospital LOS, the need for postoperative noninvasive ventilation, and the need for postoperative mechanical ventilation. They verified that none of the patients in the neostigmine group needed PNIV compared with 3% of the patients in the sugammadex group. They also verified that the incidence of POMV didn't differ between groups (3% in both groups; NA). Similarly, there was no difference in the hospital length of stay between groups (12.9 days vs. 11.4 days, respectively: NA). Togioka et al. [20] additionally evaluated patients' LOS and verified they didn't differ between groups (4.5 days in the neostigmine group vs. 4.0 days in the sugammadex group).

Berg et al. [21] study also analyzed postoperative hypoxia and the potential need for postoperative ventilation. They found that 10% of the patients in the long-acting neuromuscular blocking drugs group needed postoperative oxygen supply compared with 10.9% of the patients in the intermediate-acting group.

Baete et al. [25] study investigated whether deep neuromuscular block (NMB) puts patients at risk of postoperative respiratory impairment compared with moderate NMB. Furthermore, they evaluated the need for reintubation or noninvasive respiratory support such as CPAP or BiPAP (Bilevel positive pressure airway). They verified that the need for PNIV didn't differ between groups (6.6% vs. 3.3%, respectively; p=0.6) and that any patient needed reintubation after surgery. Like Choit et al. [17], Kabon [22] also recorded patients' LOS and verified that it did not differ between groups (7 days for both groups; p=0.45).

The definitions of postoperative pulmonary complications used in different studies are summarized in Table 4.

**Table 4:** Definitions of Postoperative Pulmonary complications PPC- Postoperative pulmonary complications

Study	Definitions
Park et al. [13].	"PPC included atelectasis (defined as a collapse of a part of the lung presented with a linear increase density on chest images), pneumonia or pulmonary edema."
Bluth et al. [14].	"PPC were defined as having occurred if any preselected complication developed within the first 5 postoperative days. The preselected complications included mild, moderate, and severe respiratory failure, acute respiratory distress syndrome, bronchospasm, new pulmonary infiltrates, pneumonia, aspiration pneumonitis, pleural effusion, atelectasis, cardiopulmonary edema, and pneumothorax."
Hemmes et al. [15].	"PPC were defined as having occurred within the first 5 days after surgery. These complications included hypoxemia, severe hypoxemia, bronchospasm, suspected pulmonary pneumonia, pulmonary infiltrate, aspiration pneumonitis, development of acute respiratory distress syndrome, atelectasis, pleural effusion, pulmonary edema caused by cardiac failure, and pneumothorax."
Karalapillai et al. [16].	"PPC were defined as having occurred within the first 7 postoperative days, including pneumonia, bronchospasm, atelectasis, pulmonary congestion, respiratory failure, pleural effusion, pneumothorax, or unplanned requirement for postoperative invasive or noninvasive ventilation."
Choi et al. [17].	"Clinical definition of PPC required a minimum of 2 criteria be documented for 2 or more days ( >48 hours ) at any time during the first 6 postoperative days: new cough/ sputum production; abnormal breath sounds compared to baseline; body temperature >38.0 °C; chest radiograph documentation of atelectasis or new infiltrate and/or physician documentation of atelectasis or pneumonia."
Squadrone et al. [18].	"Pneumonia was defined as the presence of an infiltrate on chest radiograph, plus at least one of the following, within the first month after surgery: purulent endotracheal aspirate; known pathogens on a Gram stain, or cultured from sputum or blood; temperature higher than 38.5°C or lower than 36°C, white blood cell count higher than 12 x 103/μL or less than 3.5 x 103/μL, or 20% immature forms."
Alday et al. [19].	Non applicable.
Togioka et al. [20].	"Respiratory complication as defined by the National Surgical Quality Improvement Program (postoperative pneumonia, unplanned intubation, ventilator dependency > 48 h)."
Berg et al. [21].	"PPC were defined as presence of atelectasis and pneumonia."
Kabon et al. [22].	"Postoperative morbidity is defined by a composite of major complications such as cardiac, pulmonary, infectious, gastrointestinal, renal, and coagulation complication."

Discussion

PPC is a noteworthy clinical problem in modern practice and represents a major cause of postoperative morbidity, mortality, and longer hospital stay. However, the absence of a consistent definition of postoperative pulmonary complications among studies makes it difficult to determine their frequency and clinical impact, resulting in a lack of agreement about how to treat patients to minimize the risk of PPCs [26-30]. Within this context, our systematic review attempts to fill in some gaps.

The formation of atelectasis and ventilation/perfusion mismatch has been linked to impaired pulmonary function in patients undergoing laparoscopic surgery under general anesthesia. Additionally, during laparoscopy, the use of pneumoperitoneum with carbon dioxide can accelerate atelectasis development and decrease respiratory compliance, leading to postoperative pulmonary complications and longer hospital stays. Intraoperative ventilatory strategies such as protective lung ventilation (PLV) have been implemented to enhance gas exchange during surgery. PLV involves using low tidal volume to reduce volume trauma and positive end-expiratory pressure (PEEP) to increase functional residual capacity and prevent alveolar collapse. Park et al. conducted a study comparing the use of intraoperative conventional ventilation with alveolar recruitment maneuver (ARM) to PLV. Their results indicated that the PLV strategy with low tidal volume (6 mL/kg) and 5 cm H_2_O PEEP lowered the occurrence of postoperative pulmonary complications. In particular, using the PLV strategy decreased the incidence of postoperative atelectasis and pneumonia compared to conventional ventilation (using a tidal volume of 10 ml/kg) combined with ARM (40 cm H_2_O of pressure for the 30s). Besides, according to this study, the PLV strategy resulted in fewer patients requiring noninvasive ventilation at discharge from the post-anesthesia care unit (PACU). Nevertheless, there was no difference between strategies on hospital LOS [13].

Intraoperative high levels of PEEP and alveolar recruitment maneuvers are believed to prevent lung atelectasis and decrease the occurrence of pulmonary complications. However, this approach is not without adverse effects, such as impaired hemodynamics and barotrauma. In the PROBESE trial, Bluth et al. investigated whether an intraoperative mechanical ventilation strategy using higher PEEP levels and ARM could reduce the incidence of postoperative pulmonary complications in obese patients undergoing surgery. The study found that this approach did not lead to a significant reduction in PPCs compared to a lower PEEP level without ARM. Additionally, the study found that patients in the high PEEP group were more likely to experience hypotension and bradycardia during surgery, while hypoxemia was more common in the lower PEEP group [14].

Additionally, Hemmes et al. [15] designed the PROtective Ventilation using HIgh versus LOw PEEP (PROVHILO) trial to test the hypothesis of whether a ventilation strategy with a high level of PEEP (12 cm H_2_O) plus ARM during general anesthesia protects against postoperative pulmonary complications. PROVHILO was a groundbreaking study because it was the first to integrate identical low tidal volumes (8 ml/kg) into both treatment groups, enabling the effects of high levels of PEEP to be isolated. Nevertheless, this study showed that, during mechanical ventilation with protective low tidal volumes in patients undergoing open abdominal surgery, the use of a high level of PEEP and ARM does not reduce the incidence of PPCs and is more likely to cause hemodynamic instability (that might be associated with a reduction of a venous return caused by increased intrathoracic pressure) compared with the use of low levels of PEEP (≤ 2 cm H_2_O) alone. Their findings also suggest that even though high levels of PEEP stabilize the lungs and protect against lung injury, they do not enhance lung protection during general anesthesia. As evidenced by the previous study, hypoxemia was also more common in patients randomized to the low-level PEEP group, whereas hypotension was more frequent in patients randomized to the high-level PEEP group. Nonetheless, the need for continued or new postoperative mechanical ventilation and hospital LOS stay did not differ between groups.

While supraphysiologic tidal volumes have often been used during intraoperative mechanical ventilation to prevent hypoxia and atelectasis, there has been a growing realization that this practice may actually be harmful and contribute to postoperative morbidity. Consequently, Karalapillai et al. performed a study to assess whether utilizing low-tidal-volume ventilation (6 ml/kg) could decrease the incidence of postoperative pulmonary complications during the first 7 days after major surgery in adults who received the same PEEP level (5 cm H_2_O) as those who underwent conventional-tidal-volume ventilation (10 ml/kg). Ultimately, they found that low-tidal-volume ventilation did not significantly reduce PPCs compared to conventional-tidal-volume ventilation. There were no differences in unplanned postoperative ventilation or duration of hospital stay between the two groups [16].

A lung-protective ventilation strategy with low tidal volumes effectively prevents PPC in patients undergoing major abdominal surgery. To further investigate the impact of mechanical ventilation during laparoscopic colectomy in patients with colorectal cancer, Choi et al. conducted a study where patients were randomly assigned to receive either volume-controlled ventilation (VCV) or pressure-controlled ventilation (PCV). The VCV group received a tidal volume of 8 mL/kg of ideal body weight (IBW) with no PEEP, while the PCV group initially received the same setting as VCV but switched to pressure-controlled mode with inspiratory airway pressure adjusted to deliver a tidal volume of 8 mL/kg of IBW after carbon dioxide pneumoperitoneum was induced. The study showed that postoperative respiratory complications were three times higher in the VCV group compared to the PCV group. One possible explanation for this difference may be that the pressure-controlled mode kept the airway pressure lower than the volume-controlled mode [17].

As noted, several strategies have been used to optimize oxygenation during general anesthesia. Talab and colleagues [23] study evaluated the efficacy of vital capacity maneuver (VCM), which consisted in re-expanding lung tissue with a pressure of 40 cm H_2_O maintained for 15 seconds, followed by different levels of PEEP used to prevent postoperative lung atelectasis in obese patients undergoing laparoscopic bariatric surgery. They also recorded the need for a nonbreathing O_2_ mask or reintubation. During post-anesthesia care unit (PACU) stay, they observed that fewer patients in the higher PEEP had better oxygenation, lower atelectasis scores, and lower incidence of PPC [23].

Postoperative hypoxemia complicates up to 50% of cases recovering from abdominal surgery. Even though oxygen administration and incentive spirometry are successful in treating the vast majority of these cases; respiratory failure can occur to the point where 8 to 10% of patients will require endotracheal intubation and mechanical ventilation. CPAP is well-known for improving gas exchange, minimizing atelectasis formation, and increasing functional residual capacity. Because a ventilation/perfusion mismatch due to atelectasis has been proposed to be the underlying mechanism responsible for postoperative hypoxemia, Squadrone and colleagues [18] conducted a clinical trial to compare the efficacy of CPAP with standard oxygen therapy versus standard oxygen therapy alone in the treatment of this postoperative complication. Their study verified that compared to the standard treatment, early treatment with CPAP reduced the incidence of pneumonia within the first week of surgery and reduced the need for endotracheal intubation [18].

A variety of strategies have been used to reduce the development of perioperative atelectasis; however, none of them included the period following extubation when there's a risk of airway obstruction. CPAP is commonly used to reduce the risk of airway obstruction in postoperative bariatric surgery patients. Neligan et al. conducted a study comparing immediate post-extubation CPAP, administered using a Boussignac system, with CPAP initiated in the recovery room in morbidly obese patients with obstructive sleep apnea during laparoscopic bariatric surgery and measured the incidence of postoperative reintubation. They observed that none of the patients enrolled in the study were reintubated, and so on, no difference was found between groups [24].

In the meantime, numerous studies have linked the use of neuromuscular blocking drugs with postoperative pulmonary complications, which may occur in 5% of surgeries and have a clear effect on hospital LOS and costs [20]. Acetylcholinesterase inhibitors, such as neostigmine, are widely administered to minimize the incidence of these complications. Yet, sugammadex is known for providing faster and more complete reversal than neostigmine. However, even though sugammadex ensures a higher quality reversal, the efficacy of sugammadex in preventing PPC is not so well established. Over this, Alday et al. [19] hypothesized that reversing neuromuscular block with sugammadex would result in fewer postoperative pulmonary complications than reversing with neostigmine. Still, they found no differences in the incidence of PPCs, more specifically atelectasis and pneumonia, as well as the incidence of PNIV, POMV, and hospital LOS between these two neuromuscular blocking drugs [19]. Likewise, Togioka and colleagues tested a similar hypothesis that sugammadex reduces postoperative pulmonary complications in patients aged ≥70 years having surgery ≥3 hours compared with neostigmine. The incidence of postoperative pulmonary complications defined by Canet and colleagues was one of their primary endpoints as well as hospital LOS and the proportion of patients diagnosed with respiratory complications defined by the National Surgical Quality Improvement Program - postoperative pneumonia, unplanned intubation, ventilatory dependency >48h) [20]. Their study also failed to detect a significant reduction in the incidence of PPCs and respiratory complications. Besides, hospital LOS stay did also not differ between groups. In addition, Berg et al. [21] also undertook a study that not only compared the incidence of PPCs following the use of the long-acting pancuronium or the intermediate-acting atracurium and vecuronium but also determined whether residual NMB in the recovery room is a risk factor for the development of PPC. Even though their study indicated that the use of pancuronium is a significant risk factor for the development of PPC, it did not significantly reduce the incidence of PPC when compared with atracurium and vecuronium. Similarly, the need for postoperative oxygen supply or postoperative mechanical ventilation did not differ between groups [21]. Baete and colleagues also conducted a study where they compared deep NMB, rocuronium bolus, and infusion, maintaining a post-tetanic count of 1-2, with moderate NMB, rocuronium bolus, and tops-ups to maintain a TOF count of 1-2, in laparoscopic bariatric surgery and measured the need for postoperative respiratory support. However, they found no difference between groups concerning postoperative noninvasive or mechanical ventilation [25].

Comparing crystalloids and colloids is difficult since the volume specifications for each fluid may vary. Kabon and colleagues conducted a study to test the hypothesis of whether goal-directed colloid administration during elective abdominal surgery decreases postoperative thirty-day major complications, including pulmonary complications (pulmonary embolism, pulmonary edema, respiratory failure, pneumonia, and pulmonary pleural effusion), more than goal-directed crystalloid administration. They also tested the hypothesis of whether colloid administration reduces hospital LOS. Their study revealed no difference in the incidence of postoperative complications between groups and hospital LOS. Following these assumptions, and since colloids are much more costly and have more side effects, they should be used carefully in surgical patients [22].

Limitations

This systematic review was subject to some limitations. First, even though we included studies concerning our primary and secondary outcomes, there was clear heterogeneity when it came to the definitions used, especially the PPC definition. Second, the majority of the included studies had nonsignificant results, which means that data provided little or no evidence that the null hypothesis was false, disrupting the opportunity to reach a conclusion. Lastly, we relied on a relatively limited number of databases for the identification of potentially eligible studies for our systematic review.

## Conclusions

Our systematic review found that ventilation strategies available intraoperatively and postoperatively aim to reduce the need for postoperative noninvasive ventilation (PNIV) or re-intubation with postoperative mechanical ventilation (POMV). Among these strategies, protective lung ventilation (PLV) with low tidal volume and PEEP, pressure-controlled ventilation (PCV), and postoperative continuous positive airway pressure (CPAP) with standard oxygen therapy significantly reduce the incidence of postoperative pulmonary complications. In addition, PLV with low tidal volume and PEEP and intraoperative mechanical ventilation with a vital capacity maneuver followed by 10 cm H_2_O of PEEP were found to decrease the requirement for postoperative noninvasive ventilation. CPAP with standard oxygen therapy was the only strategy that reduced the need for reintubation. However, none of the procedures reduced the hospital's length of stay.
